# Senescence-related genes as prognostic indicators in breast cancer survival

**DOI:** 10.1007/s11357-024-01384-w

**Published:** 2024-10-21

**Authors:** Zoltan Ungvari, Anna Ungvari, Monika Fekete, Csaba Kiss, Balázs Győrffy

**Affiliations:** 1 Vascular Cognitive Impairment, Neurodegeneration and Healthy Brain Aging Program, Department of Neurosurgery, University of Oklahoma Health Sciences Center, Oklahoma City, OK USA; 2 Stephenson Cancer Center, University of Oklahoma, Oklahoma City, OK USA; 3 Oklahoma Center for Geroscience and Healthy Brain Aging, University of Oklahoma Health Sciences Center, Oklahoma City, OK USA; 4 Department of Health Promotion Sciences, College of Public Health, University of Oklahoma Health Sciences Center, Oklahoma City, OK USA; 5 International Training Program in Geroscience, Doctoral College/Institute of Preventive Medicine and Public Health, Semmelweis University, Budapest, Hungary; 6 Healthy Aging Program, Institute of Preventive Medicine and Public Health, Semmelweis University, Budapest, Hungary; 7 Doctoral College, Health Sciences Program, Semmelweis University, Budapest, Hungary; 8 Department of Bioinformatics, Semmelweis University, 1094 Budapest, Hungary; 9 Cancer Biomarker Research Group, Institute of Molecular Life Sciences, HUN-REN Research Centre for Natural Sciences, 1117 Budapest, Hungary; 10 Department of Biophysics, Medical School, University of Pecs, 7624 Pecs, Hungary

**Keywords:** Aging, Gero-oncology, Senescence, Breast cancer, Survival, Prognosis, Senescent, Pharmacology

## Abstract

Breast cancer is a leading cause of cancer-related mortality among women worldwide, particularly affecting those in their later years. As the incidence of breast cancer increases with age, understanding the biological mechanisms that link aging and cancer becomes crucial. Cellular senescence, a hallmark of aging, plays a dual role in cancer by inhibiting tumorigenesis while also contributing to tumor progression through the senescence-associated secretory phenotype (SASP). This study aims to investigate the prognostic significance of senescence-related genes in breast cancer. We utilized the SenMayo gene list, a comprehensive set of senescence-related genes, to analyze gene expression data from a large cohort of breast cancer samples. The data was sourced from the Kaplan–Meier plotter, an integrated database that compiles gene expression information from multiple independent cohorts. Cox proportional hazards regression and false discovery rate (FDR) corrections were employed to evaluate the correlation between gene expression and survival outcomes, aiming to establish a prognostic signature. Our findings demonstrate that higher expression levels of senescence-related genes are significantly associated with improved survival, while lower expression levels correlate with shorter survival outcomes. These results suggest that senescence-related pathways play a protective role in breast cancer, potentially serving as valuable prognostic indicators. The identification of a prognostic signature based on senescence-related genes underscores the importance of cellular senescence in breast cancer progression and survival. Our study highlights the potential of senescence-related biomarkers in enhancing patient stratification and informing treatment strategies, contributing to the growing body of literature on the intersection of aging and cancer.

**Supplementary Information:**

The online version contains supplementary material available at 10.1007/s11357-024-01384-w.

## Introduction

Breast cancer is one of the most prevalent malignancies worldwide, particularly affecting women as they age [1, 2]. In 2022, 2.3 million women were diagnosed with breast cancer, resulting in 670,000 deaths worldwide [3]. The risk of developing breast cancer increases significantly with age, with the majority of cases diagnosed in women aged 50 years and older and the median age at diagnosis is in the early 60 s [1, 2]. This underscores its classification as an age-related disease. The rising incidence of breast cancer with advancing age highlights the need for a deeper understanding of the underlying biological mechanisms that link aging to cancer development [4]. Among these mechanisms, cellular senescence has garnered significant attention for its paradoxical role in both tumor suppression and promotion [5–8].

Cellular senescence is recognized as a hallmark of aging [7, 9–12]. Originally characterized as a state of stable cell cycle arrest in response to DNA damage-induced cellular stress, senescence acts as a crucial barrier against malignant transformation by preventing the proliferation of damaged cells [7]. On the other hand, the gradual accumulation of senescent cells over time leads to tissue dysfunction and chronic inflammation, which can drive the onset and progression of various age-related diseases, including cancer [5–7]. Moreover, the senescence-associated secretory phenotype (SASP), which involves the secretion of pro-inflammatory cytokines, growth factors, and proteases, can paradoxically promote tumor progression and influence the tumor microenvironment [6, 13, 14], further complicating the role of senescence in cancer biology.

The buildup of senescent cells across different tissues, such as the brain [15, 16], heart [17, 18], vascular system [19–23], and skin [24–26], has been linked to age-related functional decline and the pathogenesis of diseases, emphasizing the far-reaching impact of senescence on overall health. In breast cancer, the role of senescence-related genes and cellular senescence [27–63] remains an area of active investigation, particularly concerning their potential as prognostic biomarkers [59, 62]. Despite growing evidence of the involvement of senescence in cancer biology, the specific contributions of senescence-related genes to breast cancer prognosis have not been fully elucidated.

Addressing this gap, the current study aims to investigate the prognostic significance of senescence-related genes in breast cancer. Utilizing a comprehensive gene set reflective of senescence-associated pathways [9], we seek to elucidate correlations between gene expression and survival outcomes in a large cohort of breast cancer samples. By leveraging the Kaplan–Meier plotter [64, 65], an integrated database that compiles gene expression data from multiple independent cohorts, our objective is to establish a prognostic signature of senescence-related genes. We hypothesize that this signature could significantly predict survival outcomes, offering novel insights into the management of breast cancer. By focusing on the intersection of senescence pathways and breast cancer prognosis, our research aspires to contribute to the burgeoning literature on the geroscience perspective of cancer, highlighting the potential of aging-related biomarkers in enhancing patient stratification and informing treatment modalities.

## Methods

### Database setup

We conducted a comprehensive search within the GEO (https://www.ncbi.nlm.nih.gov/geo/) and EGA (https://ega-archive.org/) repositories to identify transcriptome-level gene expression datasets that also include clinical data. We focused on datasets comprising a minimum of 30 samples and selected only those generated using the Gene Expression Omnibus platforms GPL96, GPL570, and GPL571. These specific platforms were chosen because they share a set of 22,277 genes measured by identical probe sequences, ensuring consistent sensitivity, specificity, and dynamic range across samples when the same probe sets are utilized.

### Quality control and pre-processing

Each array underwent normalization using MAS5, a method previously determined to perform optimally based on comparisons with RT-PCR validated expression values [66]. MAS5 was chosen for its ability to normalize individual samples independently, ensuring that the inclusion or exclusion of any sample does not impact the overall dataset. Subsequently, a secondary scaling normalization was applied to mitigate batch effects by adjusting the mean expression of the overlapping 22,277 probes to a preset value of 1000 for each array. Only the probes from the GPL96 platform were utilized during the scaling normalization to avoid platform-specific biases resulting from the greater number of probes present in the GPL570 arrays [67].

To eliminate redundancy, we compared normalized gene expression values across all samples. In cases where identical expression values were found across multiple samples, only the dataset from the first publication was retained, with all subsequent duplicate samples removed. Five parameters were utilized for quality control: background signal, raw *Q* values, percentage of present calls, detection of bioBCD spikes, and the GAPDH/ACTB 3 to 5 ratio. Samples with positive values, or those with continuous variables falling within the 95% confidence interval across all samples, were deemed to have passed quality control. Outlier samples, characterized by failure in one parameter or more than two parameters, were identified as biased and excluded from further statistical analyses [68].

### Identification of molecular subtypes

Molecular subtypes were identified using the St Gallen criteria [69]. Due to the availability of gene expression data from all samples, we utilized these data to determine the receptor status for each patient. Specifically, a cutoff of 500 was applied to the probe set 205225_at to classify estrogen receptor positivity, while a cutoff of 4800 was used for the probe set 216836_s_at to categorize patients into ERBB2 positive or negative groups. It is important to note that the progesterone receptor was not included in this analysis due to the absence of a reliable probe set for this gene in the gene arrays.

### SenMayo senescence signature

In this study, we employed a gene set reflective of senescence-associated pathways, originally published in the study by Saul et al. [9]. This gene set, termed as SenMayo, has been confirmed to be enriched in senescent cells across different tissue types and organisms. The integrated senescence-derived gene expression signature was computed as the average expression of all included genes, and this value was used in all subsequent analyses for each tumor sample. The entire list of all genes with respectable probe sets is provided in Supplemental Table 1.

### Univariate survival analysis

Cox proportional hazards regression was performed for the entire signature which included all available genes. In order to avoid missing a potential correlation due to a specific cutoff value, we examined all possible cutoff values between the lower and upper quartiles of expression. In cases where identical *p*-values were observed, the strongest hazard ratio was selected. To address the issue of multiple hypothesis testing, the false discovery rate was controlled using the Benjamini–Hochberg method. The survival analysis focused on relapse-free survival (RFS) as the primary endpoint, as breast cancer–specific survival was not available given that most studies report either overall survival or RFS only. The optimal mean cutoff was subsequently exported into a separate database and utilized to generate Kaplan–Meier plots, which were employed to visualize the relationship between gene expression and survival.

### Multivariate analysis

Finally, we conducted multivariate Cox regression to evaluate the combined impact of the gene signature alongside other key clinical and pathological factors on relapse-free survival. The clinical parameters included in the analysis were estrogen and ERBB2 receptor status, molecular subtype, lymph node status, tumor size, and patient age. Given the substantial amount of missing data in the clinical dataset, each analysis was performed in pairs (e.g., the senescence-derived gene signature and lymph node status in a single model).

## Results

### Database setup

Not all genes of the SenMayo senescence-associated gene list were available in both array platforms. For this reason, we restricted our analysis to those samples where gene expression was determined using the GPL570 analysis platform. The whole united database includes 2006 tumor specimens with available relapse-free survival time and gene expression data at the transcriptomic level (Table 1). The average follow-up was over 61.6 months with 32.8% of the patients having a relapse event. Notably, with the exception of relapse-free survival data, not all patients had each of the clinical parameters available. The majority of patients were estrogen receptor positive (69.8%), and 22.8% were HER2-receptor positive. Of the 1380 patients with lymph node status, 58.4% were node-positive. The mean tumor size was 2.5 cm, and the mean age was 54.1 years. The detailed clinical characteristics with specific patient numbers for the entire integrated database are provided in Table 1.

**Table 1:** Clinical characteristics of the included breast cancer patients. Note that the total numbers do not add up to 100% of the patients, because with the exception of relapse-free survival data, not all patients had all data available

Feature	*n* (%)
Included datasets	14
Total number of patients	2006
Survival	
Median follow-up	54.5 months
Number of patients with an event	658
Lymph node status	
Positive	806 (58.5%)
Negative	573 (41.5%)
Age (years)	54.1 ± 12.5
Size (cm)	2.52 ± 1.42
Estrogen receptor status	
Positive	1401 (69.8%)
Negative	605 (30.2%)
HER2 receptor status	
Positive	457 (22.8%)
Negative	1549 (77.2%)
Molecular subtype	
Basal	410 (20.4%)
Luminal A	939 (46.8%)
Luminal B	462 (23.0%)
HER2 enriched	195 (9.7%)

### Univariate survival analysis

**Fig. 1:**
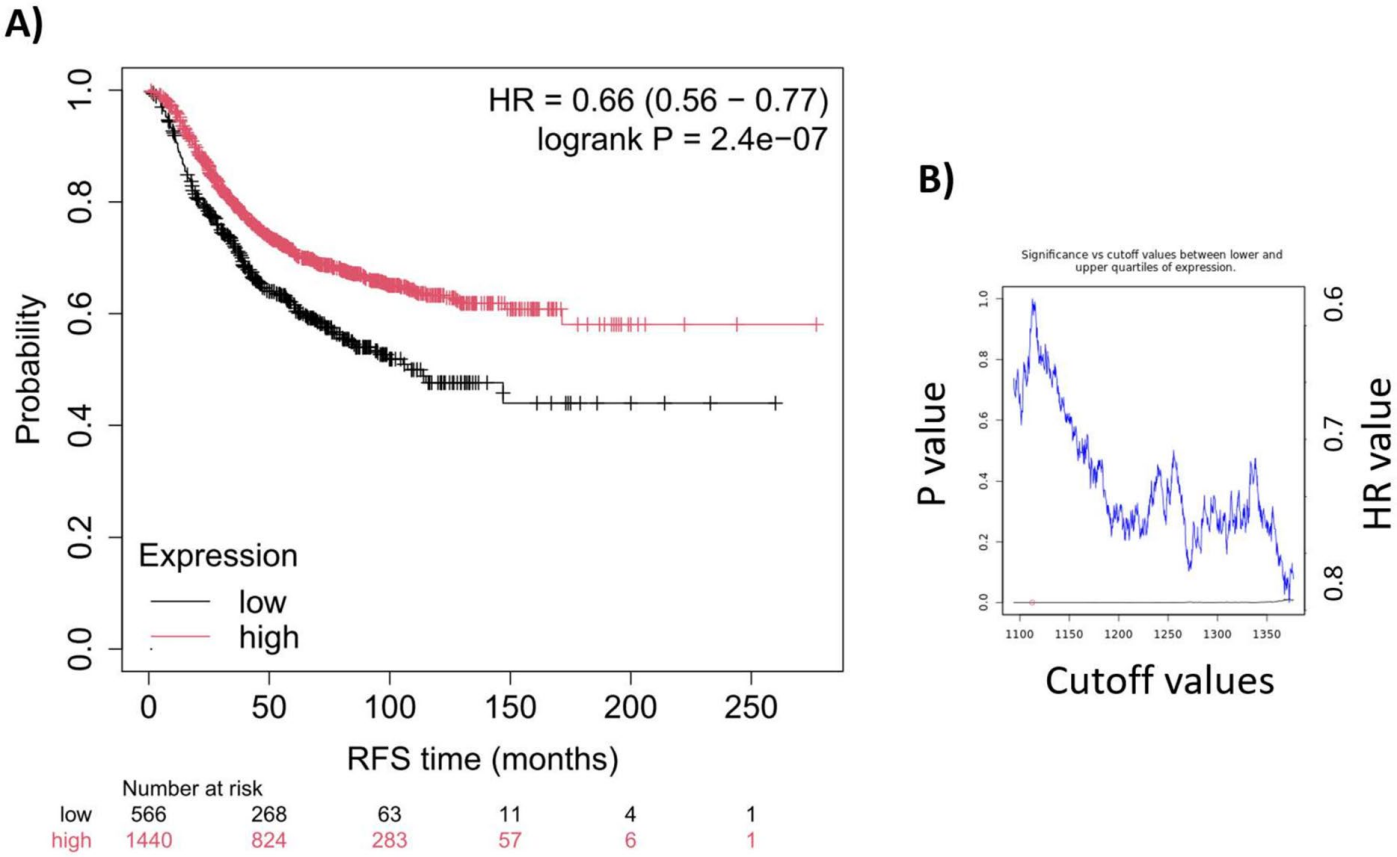
We evaluated the correlation between the senescence-derived gene signature and survival outcomes in breast cancer. Kaplan–Meier survival analysis was performed using the mean weighted gene expression of the senescence-related gene signature (**A**). The robustness of the signature was assessed across various cutoff values, as illustrated by the significance vs. cutoff plot (**B**). The lowest *p*-value, indicated by a red circle on the significance plot, was used to determine the cutoff for the Kaplan–Meier survival curve. RFS, relapse-free survival; HR, hazard rate

We established the combined signature by calculating the mean expression of all included genes, as detailed in the “Methods” section. Applying the full SenMayo senescence-associated gene list-derived signature across all available patient samples, we observed a significant correlation with relapse-free survival (HR = 0.66, 95% CI = 0.56–0.77, *p* = 2.4e-07; as illustrated in Fig. 1A). The false discovery rate was below 1%. Of note, across all cutoff values between the lower and upper quartiles of the signature’s expression, each cutoff yielded a significant *p*-value (see Fig. 1B). The median survival in the low-expression cohort was 30 months, in the high-expression cohort 46.49 months.

### Multivariate analysis

To increase the sample size available for multivariate analysis, we analyzed the signature in combination with each clinical parameter separately. The SenMayo senescence-associated gene list-derived signature remained significant when paired with lymph node status (*p* for nodal status = 4.0e-07), estrogen receptor status (*p* for ESR status = 3.1e-07), HER2 status (*p* for HER2 status = 0.0031), and size (*p* for size = 3.0e-04). Molecular subtype and age did not reach significance, but the signature itself remained significant in these two analysis settings as well. These findings indicate that the SenMayo signature possesses prognostic power independent of the available clinical and pathological parameters. In order to assess the differences in various datasets, we have run the analysis using data from the three largest cohorts independently. Notably, similar trends were observed in each of the datasets including E-MTAB-365, GSE20685, and GSE21653, but the results did not reach statistical significance (HR values of 0.77, 0.72, and 0.61, and *p*-values of 0.19, 0.16, and 0.052, respectively).

## Discussion

Our investigation into the prognostic significance of senescence-related genes in breast cancer has established a compelling link between these genes and patient survival outcomes. By utilizing the SenMayo gene list, our research underscores the potential of senescence-associated pathways as significant indicators for survival prediction in breast cancer [27, 28, 33, 38, 49, 55]. Specifically, our findings demonstrate that lower expression levels of senescence-related genes are associated with shorter survival, while increased expression correlates with longer survival. This pattern aligns with existing literature [57], reinforcing the idea that senescence processes are integral to breast cancer progression. Our approach, rooted in gero-oncology, bridges geroscience with cancer biology to offer a nuanced understanding of the complex interactions between aging mechanisms and the development and progression of breast cancer [4, 57]. This perspective is crucial for identifying novel prognostic biomarkers and enhancing patient outcomes in breast cancer treatment.

The role of senescent cells in cancer is multifaceted, involving certain senescence-related genes in both promoting and inhibiting tumor initiation, progression, and metastasis [32, 38, 39, 49, 55, 57]. Our analysis confirmed that in the context of breast cancer, lower expression of senescence-related genes is linked to poorer survival outcomes, while higher expression is associated with better survival. This finding aligns with recent studies that have identified distinct senescence phenotypes in breast tumors [33, 38, 49, 55, 57, 59], each exhibiting varying responses to therapy and implications for patient prognosis [31, 34, 37, 41–43, 45, 47, 50, 51, 61, 63]. For instance, research has demonstrated that a senescence-based scoring system can predict therapy response, with specific senescence markers correlating with improved survival outcomes [27, 28, 33, 44, 49].

Our findings, consistent with these observations, contribute to a deeper understanding of the intricate mechanisms underlying breast cancer pathogenesis. They suggest that senescence-related genes play a significant role in the disease’s progression, echoing previous studies that have shown that increased senescence in tumor cells is often associated with reduced tumor progression. However, the role of senescence varies depending on the cell type involved. For example, senescence in tumor cells typically leads to decreased proliferation, while senescence in the stroma may promote tumor progression through the secretion of SASP factors. Senescence in vascular endothelial cells [44, 46] could potentially limit angiogenesis and change the humoral milieu, further influencing tumor growth dynamics. The diverse roles of senescent cells across different compartments within the tumor microenvironment underscore the complexity of senescence in cancer biology.

The interaction between senescent cells and the tumor microenvironment, particularly through the SASP, suggests mechanisms by which senescent cells could promote tumor growth and resistance to therapy [30, 33, 37, 44, 46, 50]. While our study did not specifically investigate metastasis, it is possible that senescent cells, through their SASP, play a role in the formation of metastases [44, 62]. Further research is needed to explore the potential contributions of senescence to metastasis formation and the impact of senescence markers on therapeutic outcomes in breast cancer patients.

The role of senescence in cancer appears to vary significantly across different tumor types [70–74], potentially reflecting the diverse importance of senescence in the various cellular compartments within these tumors. In breast cancer, as our findings demonstrate, higher expression of senescence-related genes is associated with improved survival, suggesting a predominantly protective role of senescence in inhibiting tumor progression. However, this relationship is not universal across all cancers. For instance, in colorectal cancer, the expression of senescence-related genes suggests a much more complex relationship to survival [74]. In colorectal cancer, senescence may simultaneously contribute to tumor suppression in certain cellular compartments, such as within the tumor cells themselves, while also promoting tumor progression through the SASP in the surrounding stromal cells. This duality reflects the intricate and context-dependent nature of senescence, where its impact on cancer progression can be highly variable depending on the specific tumor microenvironment and the interplay between different cell types within the tumor. Such complexity underscores the need for tumor-specific studies to fully understand the prognostic implications of senescence-related genes and to develop tailored therapeutic strategies that appropriately address the multifaceted role of senescence in different cancers.

While our results corroborate the prognostic relevance of senescence in breast cancer, they also prompt further investigation into areas of discrepancy and divergence from established literature [28, 29, 33, 49, 55]. For instance, the relationship between various senescence pathways and survival outcomes warrants deeper exploration. Understanding these pathways in greater detail could help identify specific senescence-related genes that serve as reliable biomarkers for disease progression and that could be integrated into clinical practice.

This study amplifies the understanding of cellular senescence’s complex role in breast cancer pathogenesis, aligning with and extending findings from prior research. It highlights the importance of further analysis to pinpoint specific senescence-related genes that could serve as biomarkers for disease progression, offering valuable insights into the molecular pathways driving tumor growth and metastasis [59, 62, 75–80]. Such insights could be particularly useful for developing new clinical applications, including prognostic tools and therapeutic strategies.

Nevertheless, it is essential to recognize the limitations inherent in our study, including potential biases introduced by methodological factors such as variations in tumor stages, treatment protocols, and socioeconomic factors, as well as the retrospective design of the study. These limitations highlight the need for future research to integrate senescence-related gene signatures with established prognostic markers to enhance the accuracy of survival predictions in breast cancer. In our discussion, we touched on the potential roles of senescence in different tumor compartments—namely tumor cells, stromal cells, and endothelial cells. Distinguishing between the contributions of senescence in these specific compartments is indeed critical for fully understanding the role of senescence in cancer progression. Future studies will aim to investigate this by employing advanced techniques such as single-cell RNA sequencing (scRNA-seq) and spatial transcriptomics. These approaches will allow us to precisely map senescence-related gene expression in individual cell populations within the tumor microenvironment. Additionally, compartment-specific senescence markers will be used to explore how senescence in different cell types contributes to tumor growth, metastasis, and therapy resistance. This level of analysis could provide new insights into how senescence in stromal cells may promote tumor progression via the SASP, while senescence in endothelial cells may impair angiogenesis and influence vascular stability. These studies will be crucial for developing targeted therapies that address the specific roles of senescent cells in each tumor compartment.

Given the potential of senescence as a therapeutic target [5, 12, 13, 81–93], our study reinforces the need to explore interventions focused on senescence-related pathways, including strategies for inducing senescence in tumor cells or clearing senescent cells from the tumor microenvironment [30, 31, 36, 40–42, 45, 46, 51]. These approaches could offer novel therapeutic strategies to curb tumor progression and improve clinical outcomes for breast cancer patients. We recognize the importance of exploring the potential role of senescent cells in metastasis formation, particularly through the SASP. The pro-inflammatory and pro-remodeling factors secreted by senescent cells may create a microenvironment conducive to metastasis. To address this, future research will utilize breast cancer metastasis models to examine how the selective clearance of senescent cells through senolytic drugs influences metastatic spread. Additionally, it will be important to conduct proteomic and transcriptomic analyses of the SASP in metastatic niches to identify specific factors that may promote invasion and colonization of distant tissues. By integrating scRNA-seq, it will be possible to map the senescent cell populations in metastatic sites and evaluate their functional contributions to metastasis. These studies could reveal critical SASP components that drive metastatic processes, offering new targets for therapeutic intervention aimed at reducing metastasis in breast cancer.

In conclusion, our study not only highlights the prognostic value of senescence-related genes in breast cancer but also sheds light on their potential roles in the disease’s pathogenesis and progression. Future research, focused on validating these findings in larger cohorts and further dissecting the role of senescence-related mechanisms, promises to unveil novel therapeutic targets and diagnostic biomarkers, thereby advancing patient care and improving clinical outcomes in breast cancer.

## Supplementary Information

Below is the link to the electronic supplementary material.

Supplementary file1 (XLSX 13 KB)
